# Electrochemical investigation of Ti_3_C_2_T_*x*_ (MXene), N-Ti_3_C_2_T_*x*_, and the Ti_3_C_2_T_*x*_/Co_3_O_4_ hybrid composite deposited on carbon cloth for use as anode materials in flexible supercapacitors†

**DOI:** 10.1039/d4na01024h

**Published:** 2025-05-27

**Authors:** Lan Nguyen, Adnan Ali, Brahim Aissa, Sosiawati Teke, Roshan Mangal Bhattarai, Avik Denra, Oai Quoc Vu, Young Sun Mok

**Affiliations:** a Department of Chemical Engineering, Jeju National University Jeju 63243 Republic of Korea adali@jejunu.ac.kr smokie@jejunu.ac.kr; b Department of Physics, Halu Oleo University Kendari-93132 Republic of Indonesia; c Qatar Environment and Energy Research Institute (QEERI), Hamad Bin Khalifa University (HBKU), Qatar Foundation Doha 5825 Qatar

## Abstract

Supercapacitors have been studied as a potential complementary technology for rechargeable batteries, fuel cells, and dielectric capacitors. Wearable energy storage systems need freestanding, flexible electrodes for maximum functioning. Optimal energy storage system performance demands an optimal balance between mechanical component flexibility and electrode energy storage and release efficiency. This work specifically focuses on investigation and comparison of the electrochemical performance of the synthesized Ti_3_C_2_T_*x*_, N-Ti_3_C_2_T_*x*_, and the Ti_3_C_2_T_*x*_/Co_3_O_4_ hybrid composite. Co_3_O_4_ NPs have been synthesized using an innovative and cost-effective novel synthesis route employing a “microplasma discharge reactor”. This offers significant benefits, including the effective prevention of hazardous reducing agent generation in comparison to other routes. Upon exposure to 1 A g^−1^ current density, the Ti_3_C_2_T_*x*_/Co_3_O_4_ hybrid composite electrode demonstrates a maximum gravimetric capacity of 128 F g^−1^ and a specific capacitance of 576.7 F g^−1^, exhibiting a significant 95.06% increase in specific capacitance compared to Ti_3_C_2_T_*x*_. Furthermore, from the kinetic analysis of the CV curves, it has been noticed that the contributions of the diffusion-controlled and pseudocapacitive-controlled processes are 60% and 40%, respectively, in the charge storage for the applied Ti_3_C_2_T_*x*_/Co_3_O_4_ hybrid composite electrode.

## Abstract 2

Supercapacitors have been studied as a potential complementary technology for rechargeable batteries, fuel cells, and dielectric capacitors.

## Graphical abstract

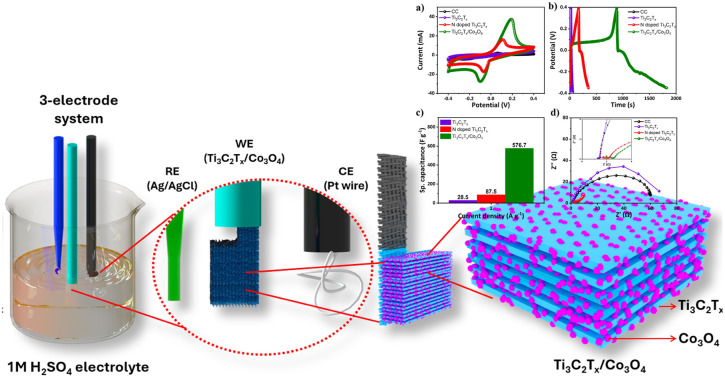

## 1. Introduction

In today's world, the growing concern over global warming has sparked interest among researchers worldwide in the development of eco-friendly materials and innovative devices. A diverse array of energy storage devices are available to cater to different requirements, including supercapacitors, batteries, fuel cells, solar cells, wind energy devices, and hydropower devices. These technologies provide various solutions for energy storage needs.^1–3^ Out of these technologies, wind energy and solar energy rely on weather and sunlight, presenting certain limitations.^4,5^ In some cases, when it comes to power requirements, supercapacitors are the most practical option^6^ because supercapacitors have an edge in energy, power density, lifespan, safety, and durability.^7^ Supercapacitors possess impressive qualities such as high specific capacitance values, quick charge–discharge characteristics, and promising storage capacity for numerous applications.^8^ Supercapacitors are categorized into two types: electric double-layer capacitors (EDLCs) and pseudocapacitors. In EDLCs, carbon resources such as graphene or carbon nanotubes are commonly utilized as electrode materials.^9^ On the other hand, pseudocapacitors rely on redox reaction processes.^10^ Pseudocapacitors have garnered significant interest for their impressive specific capacitance and rapid redox kinetics.^11^ The exchange of ions between the electrode material and the electrolyte leads to the storage of charge in the electrode. This deposition of ions on the electrode surface affects electrochemical performance due to their physical and chemical characteristics.^12,13^ Thus, the selection of the electrode material plays a critical role in determining the electrochemical properties of a supercapacitor, leading scientists to explore a wide variety of materials in order to get optimal capacitance results.^14,15^ Recent investigations by researchers have focused on materials, such as metal oxides, metal–organic frameworks (MOFs), and two-dimensional (2D) materials such as graphene, MXenes, and their composites.^16^ Notably, MXenes, which include carbonitrides, nitrides, and 2D transition metal carbides (TMCs), have garnered significant attention due to their remarkable electrical conductivity, mechanical properties, and surface functionalities.^17–20^ Supercapacitors have shown promising results with Ti_3_C_2_T_*x*_ (MXene) applied as an electrode. The flexible surface chemistry and high conductivity of Ti_3_C_2_T_*x*_ enable the efficient transport of electrolyte ions.^15,21–23^ The presence of multiple functional groups in Ti_3_C_2_T_*x*_ allows it to demonstrate hydrophilic behavior.^24–28^

Studies have explored the integration of transition metal oxides and sulfides with MXene-based supercapacitors to enhance their energy storage capabilities.^29–34^ Among the metal oxides, the face-centered cubic compound Co_3_O_4_ has gained a lot of attention for its possible use as an electrode material in energy storage devices.^11,35–38^ It has been demonstrated that, in comparison to bulk materials, nanoscale materials with small particle size and a high surface/volume ratio can increase the number of active sites, decreasing the mass and charge diffusion distances, which improve electrochemical performances.^39,40^ The electrochemical properties of Co_3_O_4_ have garnered significant interest from researchers. Its mixed-valence state and spinel structure facilitate ion diffusion, leading to improved stability and electrochemical performance over CoO in supercapacitor applications. The redox reactions of the Co^3+^/Co^2+^ pair are pivotal to pseudocapacitance, which is responsible for the high specific capacitance of Co_3_O_4_.^41–43^ Co_3_O_4_ nanoparticle synthesis *via* hydrothermal,^44^ sol–gel,^45^ solvothermal,^46^ spray pyrolysis,^47^ microemulsion,^48^ biological templating,^29^ and electrodeposition methods has been reported.^29^ In this research, Co_3_O_4_ nanoparticles (NPs) have been synthesized using a novel device called the microplasma discharge reactor (MPR). MPR is a direct, cost-effective, and reliable device that functions at standard atmospheric pressure and produces nanoparticles with outstanding uniformity.^49^ The interlayer spacing in Ti_3_C_2_T_*x*_ nanosheets and the presence of surface terminations, such as functional groups, along with the large surface area of Co_3_O_4_ nanoparticles, contribute to their superior ability to enhance charge transfer and redox reaction kinetics. Consequently, the potential for numerous redox reaction sites leads to enhanced electrochemical performance. In this work, we have adopted the hybridization approach using Co_3_O_4_ nanoparticles coupled with Ti_3_T_2_C_*x*_ to enhance the stability of MXene-based electrodes while concurrently maintaining their exceptional electrochemical properties. Herein, Ti_3_C_2_T_*x*_, N-Ti_3_C_2_T_*x*_ and the Ti_3_C_2_T_*x*_/Co_3_O_4_ hybrid composite have been successfully synthesized. Co_3_O_4_ synthesized using the MPR was incorporated in between and on the outer surfaces of the 2D-Ti_3_C_2_T_*x*_ nanosheets for energy storage application in supercapacitors. The structure, morphology, and composition of the synthesized materials were thoroughly investigated using scanning electron microscopy (SEM), energy dispersive spectroscopy (EDS), X-ray diffraction (XRD) analysis, Raman spectroscopy, transmission electron microscopy (TEM) and X-ray photoelectron spectroscopy (XPS), while their electrochemical performance was tested on an AutoLab PGSTAT204N, Metrohm electrochemical workstation in a three-electrode configuration system by measuring the Cyclic Voltammetry (CV), Galvanic Charging/Discharging (GCD), and Electrochemical Impedance Spectroscopy (EIS) profiles. For investigating the role of functional groups, FTIR spectra of Ti_3_C_2_T_*x*_, Co_3_O_4_ and the hybrid nanocomposite Ti_3_C_2_T_*x*_/Co_3_O_4_ were obtained. It has been observed through thorough investigation that the Ti_3_C_2_T_*x*_/Co_3_O_4_ hybrid composite performance as an anode is the best compared to Ti_3_C_2_T_*x*_ and N-Ti_3_C_2_T_*x*_.

## 2. Experimental

### 2.1. Materials and preparation of the multilayer Ti_3_C_2_T_*x*_ MXene

MAX phase titanium aluminum carbide (Ti_3_AlC_2_) was synthesized by mixing Ti_2_AlC (Sigma Aldrich) and TiC in a 1 : 1 mole ratio. The mixture was ball-milled for 24 hours. After ball milling, the mixture was sintered for 2 hours at 1300 °C in argon at 50 sccm. The tiny Ti_3_AlC_2_ pellet was cooled to room temperature. The material was carefully milled and sifted. Ti_3_AlC_2_ powder with a particle size of ≤40 μm was made using this process. Selective etching of Ti_3_AlC_2_ using HF (Fisher Scientific). Over 5 minutes, 2 g of sieved Ti_3_AlC_2_ was added to a 20 milliliter solution of 48–51% HF. The mixture was stirred at 250 rpm with a magnetic stirrer on a hot plate at 40 °C for 72 hours. The reaction mixture was washed with deionized water (DIW) by centrifugation at 3500 rpm for 5 minutes each time. After each spinning, the acidic supernatant was separated and additional DIW was added and centrifuged. At pH 5, the process stopped. The whole procedure produced multilayer MXene (m-Ti_3_C_2_T_*x*_) flakes. The solution was vacuum-filtered to extract m-Ti_3_C_2_T_*x*_ using a Celgard®3501 polypropylene membrane with a thickness of 25 μm and pore size of 0.064 μm. The powder was furnace-dried at 80 °C under normal conditions.

### 2.2. Preparation of exfoliated Ti_3_C_2_T_*x*_ MXene (d-Ti_3_C_2_T_*x*_) from m-Ti_3_C_2_T_*x*_

A 300 mg sample of finely ground multilayer Ti_3_C_2_T_*x*_ powder was added with 50 mL of degassed deionized water. The mixture was then sonicated for one hour using a Cole Parmer 750 watt ultrasonic homogenizer, set at 65% amplitude with an on–off cycle of 5 seconds on and 2 seconds off. During sonication, a steady argon flow of 10 sccm was maintained, and the container was kept in an ice bath to prevent temperature rise and potential oxidation of Ti_3_C_2_T_*x*_. Following sonication, the solution underwent centrifugation at 3500 rpm for 30 minutes to separate the d-Ti_3_C_2_T_*x*_ flakes. The resulting supernatant was then frozen at −50 °C for 20 minutes and subsequently freeze-dried overnight using a freeze dryer system (FDTE-8012).

### 2.3. Preparation of nitrogen doped Ti_3_C_2_T_*x*_, MXene (N-Ti_3_C_2_T_*x*_)

To synthesize N-Ti_3_C_2_T_*x*_, a solution was prepared by adding 100 mg of Ti_3_C_2_T_*x*_ in 50 mL of deionized water. Subsequently, a 5 mg quantity of urea was introduced into the mixture while maintaining constant stirring. The mixture was transferred to a hydrothermal Teflon-lined autoclave unit and underwent a reaction with urea at a temperature of 180 °C for a duration of 18 hours. Subsequently, the autoclave underwent a cooling process until it reached the ambient temperature, resulting in nitrogen doped Ti_3_C_2_T_*x*_. The N-Ti_3_C_2_T_*x*_ was subjected to several DIW washes and then centrifuged at a speed of 5000 rpm for a duration of 5 minutes. The N-Ti_3_C_2_T_*x*_ sediment was subjected to freezing at a temperature of −55 °C for a duration of 24 hours. Subsequently, it was subjected to lyophilization using an FDTE-8012 freeze dryer to remove the ice.

### 2.4. Preparation of cobalt oxide nanoparticles (Co_3_O_4_ NPs)

**Fig. 1:**
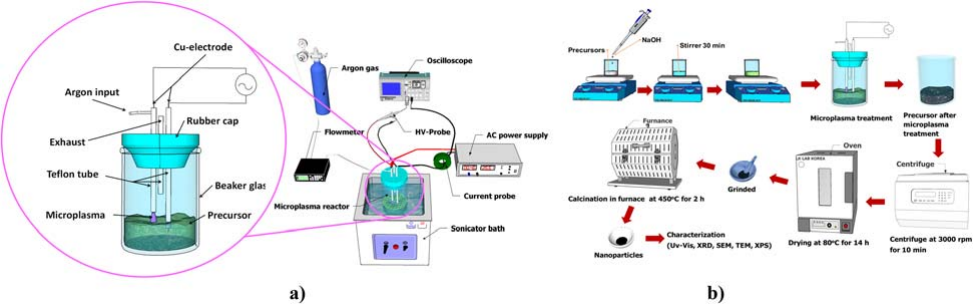
(a) Schematic diagram of the MPR setup and (b) synthesis process of cobalt oxide nanoparticles.

Cobalt oxide nanoparticles were prepared by an economic technique, microplasma reactor discharge.^49^ A 0.01 M solution of (Co(NO_3_)_2_·6H_2_O) was added to 25 mL of DIW. Then, 1 M NaOH was introduced into the pH = 10 solution. A magnetic stirrer was used to agitate the mixture for 30 minutes at 100 rpm in an ambient environment. The sample was placed in an ultrasonication bath set at a frequency of 28 kHz and treated with plasma for 25 minutes. After centrifuging, the resultant solution was cleaned with ethanol and allowed to dry for 14 hours at 80 °C in an oven. Ultimately, the powder was ground and then air-fired for two hours at 450 °C (3.5 °C per minute) in a furnace. The step-by-step process of generating Co_3_O_4_ NPs is schematically shown in Fig. 1. Detailed explanation of the MPR mechanism of producing nanoparticles is given in the ESI,† and the mechanism is schematically shown in Fig. S1(a).† The size controlled Co_3_O_4_ nanoparticle synthesis *via* the MPR synthesis technique and particle size distribution analysis are given in Fig. S1(b).† Additionally, in the ESI,† the cost estimation calculation per gram Co_3_O_4_ nanoparticles *via* the MPR technique is provided, which is $7 per gram.

In this step, the Ti_3_C_2_T_*x*_/Co_3_O_4_ hybrid composite was synthesized by adding 20 mg of Ti_3_C_2_T_*x*_ and 5 mg of Co_3_O_4_ in 30 mL of DIW. The mixture underwent sonication in a bath for 30 minutes at room temperature. Following bath sonication, the mixture was placed in a Teflon hydrothermal unit. The hydrothermal unit was placed in a furnace and heated to 180 °C for a duration of 18 hours. It was then allowed to cool down to ambient room temperature. The resulting dispersion was centrifuged at a speed of 5000 rpm for a duration of 3 minutes, and this procedure was repeated a total of 4 times. The sediment was subsequently dried at 80 °C for 8 hours. After that, the dried powder of Ti_3_C_2_T_*x*_/Co_3_O_4_ was ground in a mortar and then subjected to calcination in an argon environment at 400 °C for 2 hours.

## 3. Preparation of electrodes

**Fig. 2:**
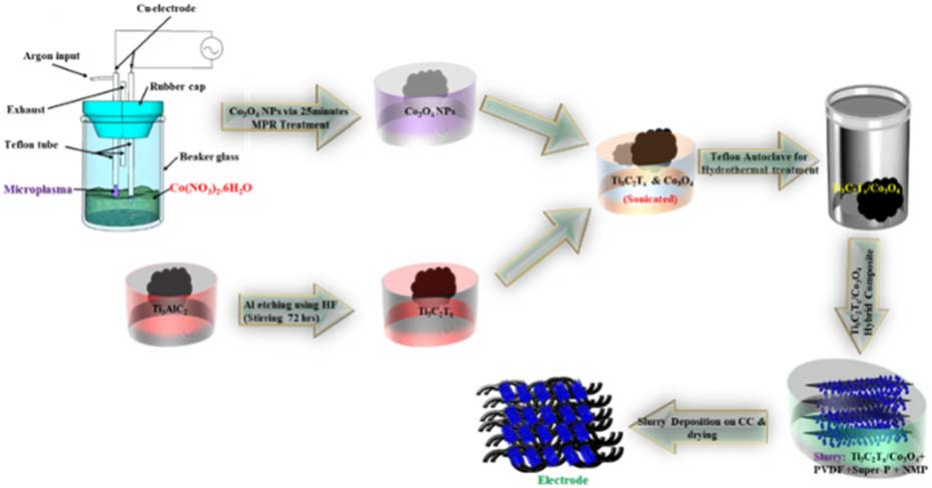
Schematic diagram of Ti_3_C_2_T_*x*_/Co_3_O_4_ hybrid composite deposition on carbon cloth.

Before applying the active material mixture as the electrode on carbon cloth (CC), it went through a 10 hour drying process at 80 °C, and after it was treated with HNO_3_, rinsed with DIW, ethanol, and acetone, and then desiccated. The anode electrode material was prepared by combining Ti_3_C_2_T_*x*_, N-Ti_3_C_2_T_*x*_ and the Ti_3_C_2_T_*x*_/Co_3_O_4_ hybrid composite one by one with Super P and polyvinylidene fluoride (PVDF) in a proportion of 8 : 1 : 1.5 drops of *N*-methyl-2-pyrrolidone (NMP) was added to the mixture to prepare the slurry. It was then ground for 30 minutes in a mortar. After evenly applying the electrode material onto a 2.25 cm^2^ section of the CC substrate, it underwent a thorough drying process for 8 hours at 100 °C in a vacuum furnace. The loaded active mass was approximately 5.6 mg. Fig. 2 presents a detailed schematic diagram showcasing the various steps in the synthesis of the Ti_3_C_2_T_*x*_/Co_3_O_4_ hybrid composite. This includes the preparation of the slurry and the subsequent deposition process.

## 4. Materials characterization

The morphology analysis was conducted using field emission scanning electron microscopy (FE-SEM) with a TESCAN MIRA3. The chemical compositions were evaluated through elemental mapping (TESCAN, MIRA3) and energy-dispersive spectroscopy (EDS) at 15 kV. The XRD technique was utilized to investigate the structure of synthesized Ti_3_C_2_T_*x*_ (unexfoliated & exfoliated), Co_3_O_4_ NPs, and the Ti_3_AlC_2_/Co_3_O_4_ hybrid composite. A PANanalytical Empyrean XRD instrument was employed, using Cu Kα1 radiation at *λ* = 0.15406 nm. The scan range for this investigation was 2*θ* values of 5° to 60°. To accurately determine the elemental composition, chemical state, and electronic structure of the materials, a Thermo Fisher Scientific Theta Probe K-ALPHA + XPS system using monochromatic Al Kα radiation with a wavelength of 1486.6 eV at 12 kV at the KBSI Busan Center was utilized. Additionally, Raman spectroscopy and FTIR characterization experiments were carried out to gain a better understanding of the role of structure and functional groups in energy storage. For the electrochemical analysis, a three-electrode system was employed, which comprised an Ag/AgCl reference electrode, a platinum wire counter electrode, and a working electrode prepared according to the method outlined in the Preparation of electrodes section, all immersed in 1 M H_2_SO_4_. The performance of the electrode materials was assessed using an AutoLab PGSTAT204N Metrohm electrochemical workstation, employing techniques such as cyclic voltammetry (CV), galvanostatic charge–discharge (GCD), and electrochemical impedance spectroscopy (EIS).

## 5. Results and discussion

### 5.1. Microstructure and phase analysis

**Fig. 3:**
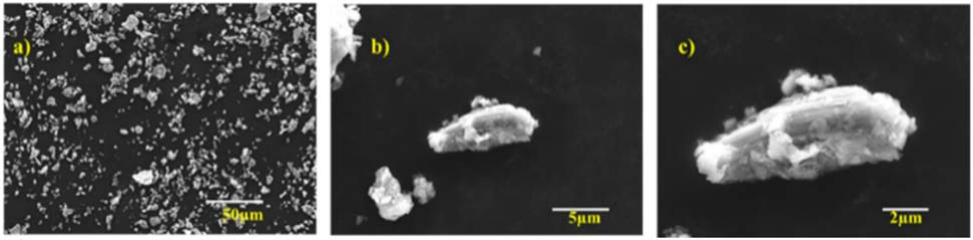
Low to high resolution FESEM images of Ti_3_AlC_2_ (a)–(c).

The morphology of Ti_3_AlC_2_ was analyzed using FE-SEM. As depicted in Fig. 3 (a–c) and S2,† the Ti_3_AlC_2_ particles are significantly smaller than 40 μm, which is beneficial for selectively etching away Al from the MAX phase within an optimized minimal duration.

**Fig. 4:**
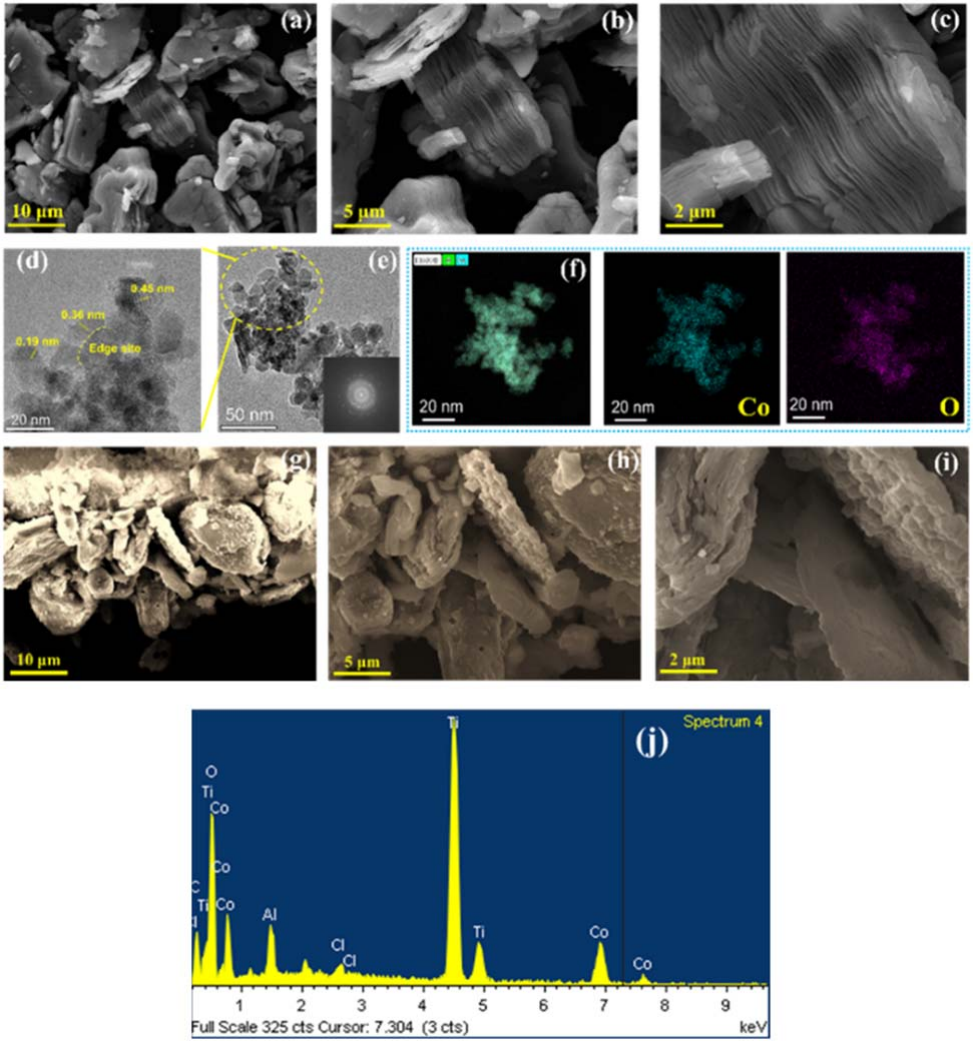
(a)–(c) FE-SEM images of Ti_3_C_2_T_*x*_, (d) HRTEM micrograph of Co_3_O_4_ NPs, (e) TEM image of Co_3_O_4_ NPs with the corresponding FFT shown in the inset, (f) HAADF-EDS color mapping of Co NPs showing Co and O elements, (g)–(i) FE-SEM images of the Ti_3_C_2_T_*x*_/Co_3_O_4_ hybrid composite, (j) EDS analysis of the Ti_3_C_2_T_*x*_/Co_3_O_4_ hybrid composite.

The etched Ti_3_AlC_2_ is depicted in Fig. 4(a–c) and S3† through a series of FESEM images that vary from low to high resolution. After the etching process, the weakly bonded Al is almost etched away, resulting in unexfoliated, stacked flakes of Ti_3_C_2_T_*x*_. The process of selectively etching Al from Ti_3_AlC_2_ to produce Ti_3_C_2_T_*x*_ presents a series of intricate challenges. The etchant employed here was 48% HF. Residual Al atoms still exist within the Ti_3_C_2_T_*x*_ framework, potentially as a result of incomplete chemical reactions or intrinsic physical barriers associated with the layered architecture. The surface termination groups, including –OH, –F, or –O, incorporated during the etching process may interact with residual Al, thereby complicating its thorough elimination. Probe sonication was conducted under an argon flow to prevent oxidation and to enhance the interlayer spacing among the flakes. In Fig. S4,† lattice spacing increase of Ti_3_C_2_T_*x*_ is schematically shown after Ti_3_AlC_2_ etching. High-resolution transmission electron microscopy (HRTEM) images of Ti_3_C_2_T_*x*_ are shown in Fig. S5,† which show the few layers and multilayers of Ti_3_C_2_T_*x*_. HRTEM images of Co_3_O_4_ nanoparticles synthesized using the microplasma discharge reactor are given in Fig. 4(d)–(e). Distinct lattice planes at approximately 0.45, 0.36, and 0.19 nm have been discerned upon examination. The crystalline structure within the Co_3_O_4_ nanoparticle is confirmed by the fast Fourier transform (FFT) image shown in Fig. 4(e) inset. The Co_3_O_4_ nanoparticle (NP) sample size is definitively smaller than 15 nm. Fig. 4(f) presents the color mapping images of the nanobulk, corresponding to the high-angle annular dark-field energy-dispersive spectroscopy (HAADF-EDS) results, highlighting the presence of oxygen and cobalt in the Co_3_O_4_ NP sample. EDS analysis results of Ti_3_C_2_T_*x*_ are given in Fig. S6.†Fig. 4(g)–(i) display images of the Ti_3_C_2_T_*x*_/Co_3_O_4_ hybrid composite at varying resolutions, from low to high. The FESEM analysis indicates that the Co_3_O_4_ nanoparticles have been successfully intercalated among the Ti_3_C_2_T_*x*_ flakes due to their size being smaller than 15 nm. The integration is designed to prevent the restacking of Ti_3_C_2_T_*x*_ flakes, thereby preserving their conductivity and high specific surface area. This contributes significantly to the properties of the hybrid composite, which is advantageous for redox reactions in electrochemical analysis. Fig. 4(j) shows the EDS spectrum of the hybrid composite Ti_3_C_2_T_*x*_/Co_3_O_4_, which confirms the presence of Ti, Co, C, O, Cl, and Al peaks among others, which is an indication that it is not 100% etched away. The EDS mapping of the hybrid composite Ti_3_C_2_T_*x*_/Co_3_O_4_ is given in Fig. S7.† It is obvious that in the hybrid composite there are two significant peaks at around 4.5 keV and 4.9 keV corresponding to Ti. Its prominent sharp peaks with high intensity indicate that the Ti concentration is high. Similarly, three distinct peaks corresponding to Co in the EDS spectrum of the hybrid composite appeared at 6.9 keV and 7.4 keV, and a lesser intensity peak appeared around 0.8 keV. The intensity of the Co peaks suggests that it is present in significant quantities, but the titanium content is more. There is a noticeable peak at around 0.5 keV in the EDS spectrum of O. This is because, in the hybrid composite, Co is present as oxide, and a small amount of Ti_3_C_2_T_*x*_ is converted to TiO_2_ as well. Oxygen is present,^50^ however, at a lower concentration, as evidenced by the peak height that is smaller than that of Ti and Co. Cl and Al impurities supplement the main components of the hybrid composite, which also include Ti, Co, C, and O. Also, these results are supported by the XPS and XRD analysis results of the hybrid composite.

### 5.2. Structural analysis *via* X-ray diffraction

**Fig. 5:**
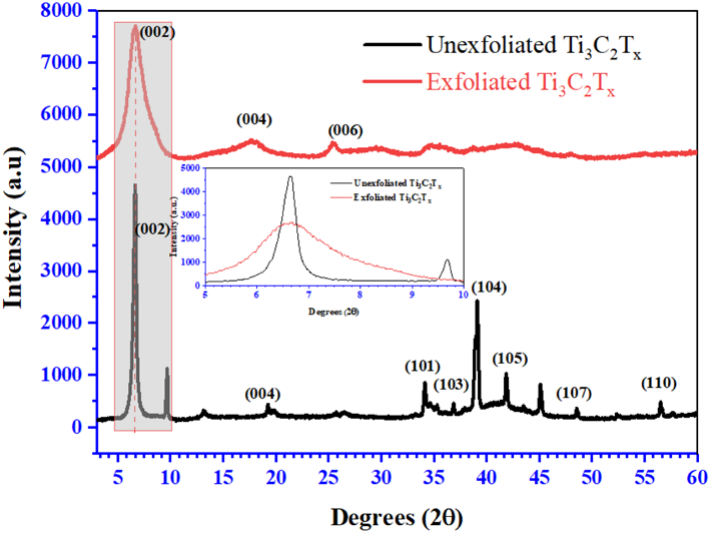
Unexfoliated and exfoliated Ti_3_C_2_T_*x*_ XRD patterns.

The XRD patterns for Ti_3_C_2_T_*x*_, both unexfoliated and exfoliated, are presented in Fig. 5. The diffraction peak positions of MAX correspond well with the simulation based on standard data (JCPDS no. 00-052-0875). Observations indicate that in unexfoliated Ti_3_C_2_T_*x*_ the corresponding peaks are narrower. However, after the exfoliation of Ti_3_C_2_T_*x*_, these signature peaks broaden, and their intensities decrease. Following chemical exfoliation with HF, the (002) peak of Ti_3_C_2_T_*x*_ broadens and shifts to a lower angle of 6.2° from its original position of 9.4°, and the diffraction peak disappears, confirming the transformation of the MAX (Ti_3_AlC_2_) phase into the MXene (Ti_3_C_2_T_*x*_) phase. Similarly, the diffraction peak positions of Co_3_O_4_ nanoparticles corresponded well with the simulation based on standard data (JCPDS no. 00-043-1003). Further on, XRD analysis of the Ti_3_C_2_T_*x*_/Co_3_O_4_ hybrid composite shows the (002) diffraction peak which is shifted further to 2*θ* = 5.7°, signifying an increase in interlayer spacing. This is due to the insertion of Co_3_O_4_ nanoparticles between the Ti_3_C_2_T_*x*_ layers. For more deep structural investigation and insight, Raman spectroscopy was carried out to support the findings of the XRD analysis. Raman spectroscopy analysis provides detailed information about the chemical structure of Ti_3_C_2_T_*x*,_ CO_3_O_4_ and the hybrid composite Ti_3_C_2_T_*x*_/Co_3_O_4_, and the spectra are presented in Fig. S9.†

### 5.3. Structural and compositional analyses *via* XRD and XPS

**Fig. 6:**
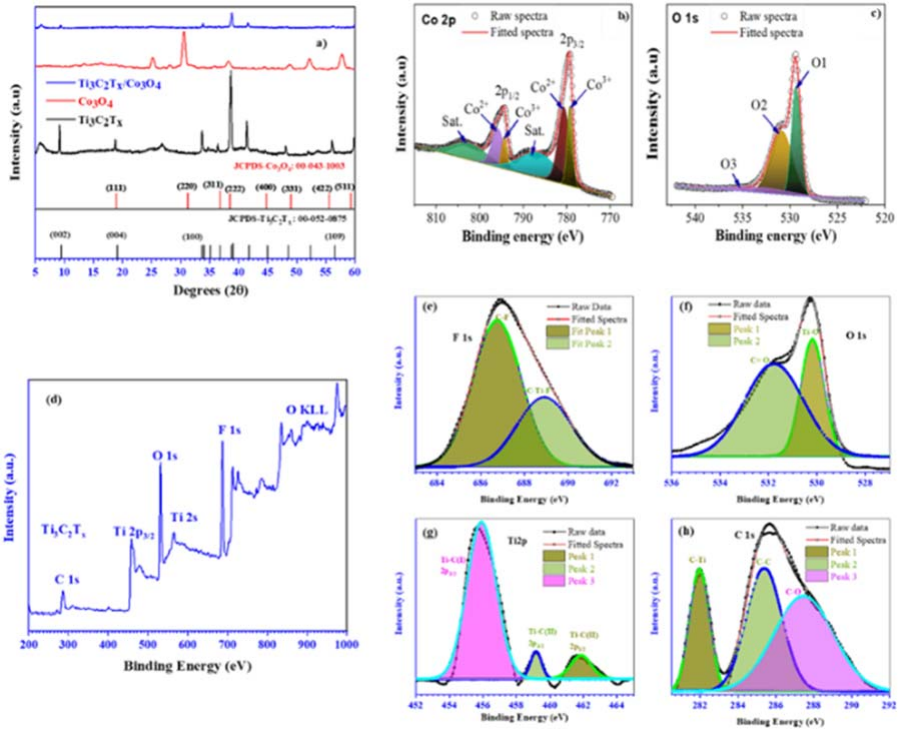
(a) XRD patterns of Ti_3_C_2_T_*x*_, Co_3_O_4_ NPs, and the Ti_3_C_2_T_*x*_/Co_3_O_4_ hybrid composite, (b) and (c) XPS spectra of Co_3_O_4_ core levels, (d), (e)–(h) XPS survey and core level spectra of F 1s, O 1s, Ti 2p and C 1s respectively.

XRD analysis was conducted for hydrothermally synthesized N-Ti_3_C_2_T_*x*_ and Ti_3_C_2_T_*x*_/Co_3_O_4_ hybrid composites from exfoliated Ti_3_C_2_T_*x*_ and MRP synthesized Co_3_O_4_ nanoparticles to examine and contrast the structural alterations, as shown in Fig. 6(a).^15^ The XRD pattern of the hybrid composite indicates the characteristic peaks of both Ti_3_C_2_T_*x*_ and Co_3_O_4_ phases. A subtle shift toward lower angles was observed in the peaks, and some peaks overlapped and broadened in the XRD pattern of the Ti_3_C_2_T_*x*_/Co_3_O_4_ composite. This indicates that the Co_3_O_4_ nanoparticles were effectively incorporated into the Ti_3_C_2_T_*x*_ nanosheets.^11,49^ The XRD pattern of N-Ti_3_C_2_T_*x*_ is given in Fig. S8(a).†

XPS analysis was conducted on the synthesized samples to examine their chemical states and composition. In Co_3_O_4_, the binding energy (BE) peaks of Co 2p_3/2_ and Co 2p_1/2_ can be observed in the range of 779.38 to 779.68 and 794.38 to 794.68 eV, respectively, as shown in Fig. 6(b). The 2p binding energies for CoO and Co_3_O_4_ spinel phases are the same, which poses a challenge in distinguishing between the two. Energy separation (Δ*E*) between the Co 2p_3/2_ and Co 2p_1/2_ peaks amounts to 15.0 eV for Co_3_O_4_ and 16.0 eV for CoO. This 1.0 eV difference is significant as it distinguishes CoO from Co_3_O_4_. Apart from that, the two shake-up satellites distinguish the Co_3_O_4_ spinel phase.^51,52^Fig. 6(c) shows the XPS analysis of O 1s. In the context of nanoparticle materials used in catalysis, energy conversion/storage, and sensors, acknowledging the presence of oxygen vacancies is crucial.^53,54^ The O 1s spectrum reveals a peak for O1, which signifies the presence of metal oxides (Co–O), within a binding energy range from 529.18 to 529.48 eV. An additional peak for O2 suggests the existence of oxygen vacancies, with a binding energy between 530.68 and 530.88 eV. The O3 peak is a prominent feature indicating the presence of surface hydroxyl molecules (Co–OH) with a binding energy between 533.48 and 534.68 eV. The presence of the O1 peak in the XPS profiles confirms the successful synthesis of Co_3_O_4_ using the microplasma discharge reactor.^49,55,56^Fig. 6(d) presents the XPS survey spectra of the etched and exfoliated Ti_3_C_2_T_*x*_. The analysis of the XPS spectra for Ti_3_C_2_T_*x*_ required the application of curve fitting to derive pertinent information. The bonding peaks within the F 1s, O 1s, Ti 2p, and C 1s XPS spectra have been precisely identified through meticulous analysis.^57,58^

The exfoliated layers of Ti_3_C_2_ are coated with terminal species and adsorbates on their surfaces. Distinguishing termination species from adsorbates is essential, as they significantly impact the synthesis of exfoliated Ti_3_C_2_ layers. Terminal species function analogously to the Al layer in Ti_3_AlC_2_, serving comparable roles in the material's structure and properties. Understanding the role of terminal species is crucial for creating appropriate conditions for electron redistribution in Ti_3_C_2_ layers and enhancing the strength of Ti–C covalent bonds.^57^Fig. 6(e) displays the F 1s spectrum obtained from the termination species present on the surface of Ti_3_C_2_T_*x*_. Considering the variance in slope between the low and high binding energy sides, it becomes evident that the fitting curve must accommodate two distinct peaks. Two distinct peaks were detected; one at 686.7 eV, signifying the presence of C–F bonds, and another at 688.94 eV, indicative of C-Ti–F bonds.^59,60^Fig. 6(f) illustrates the O 1s spectrum, which is characterized by two distinct peaks. A meticulous examination of the ascending trend on the lower binding energy side reveals a solitary bonding peak at 530.1 eV, suggestive of a Ti–O bond. The peak at 531.79 eV overlaps with the one at 530.1 eV, associated with C=O, obscuring the observation of the rising gradient on the lower binding energy side of the peak.^59,61^Fig. 6(g) displays the Ti 2p spectrum. Curve fitting of this spectrum can be quite complex due to the presence of two XPS peaks, 2p_3/2_ and 2p_1/2_, which exhibit different intensities. The peak at 455.9 eV is less intense than the peak at 461.63 eV. A notable difference of approximately 6.0 eV in binding energy between these two peaks simplifies the curve fitting process. Additionally, there is a peak at the higher binding energy side of Ti 2p_3/2_, which does not exhibit any additional features.^57,58^Fig. 6(h) displays the C 1s XPS spectrum. The peak observed at 282.0 eV within the C 1s spectrum suggests the presence of carbide materials, particularly Ti–C. Additionally, peaks at binding energies of 285.66 eV and 287.65 eV are present, corresponding to C–C and C–O bonds, respectively. The latter peaks, found at higher binding energies, are typically linked to graphite.^62^ Due to the multi-stage synthesis process of exfoliated Ti_3_C_2_T_*x*_, there is a high likelihood of contamination and the presence of residual un-etched Ti_3_AlC_2_. From the C 1s spectrum, it is evident that the peak associated with carbide materials is distinct from any contaminations or impurities. There is a noticeable difference in the steepness of the rising slope on the low BE side compared to the high BE peaks.^58^ For N-Ti_3_C_2_T_*x*_, XPS analysis results are given in Fig. S8(b),† and it has been observed that doped nitrogen has bonded with Ti as well as with C and O present in Ti_3_C_2_T_*x*_.

## 6. Electrochemical characterization by CV, GCD, and EIS

The electrodes Ti_3_C_2_T_*x*_, N-Ti_3_C_2_T_*x*_ and Ti_3_C_2_T_*x*_/Co_3_O_4_ deposited on CC were thoroughly analysed to investigate their electrochemical performance. For this, cyclic voltammetry (CV), galvanostatic charge–discharge (GCD), and electrochemical impedance spectroscopy (EIS) using a three-electrode system in a 1 M H_2_SO_4_ electrolyte have been performed.

**Fig. 7:**
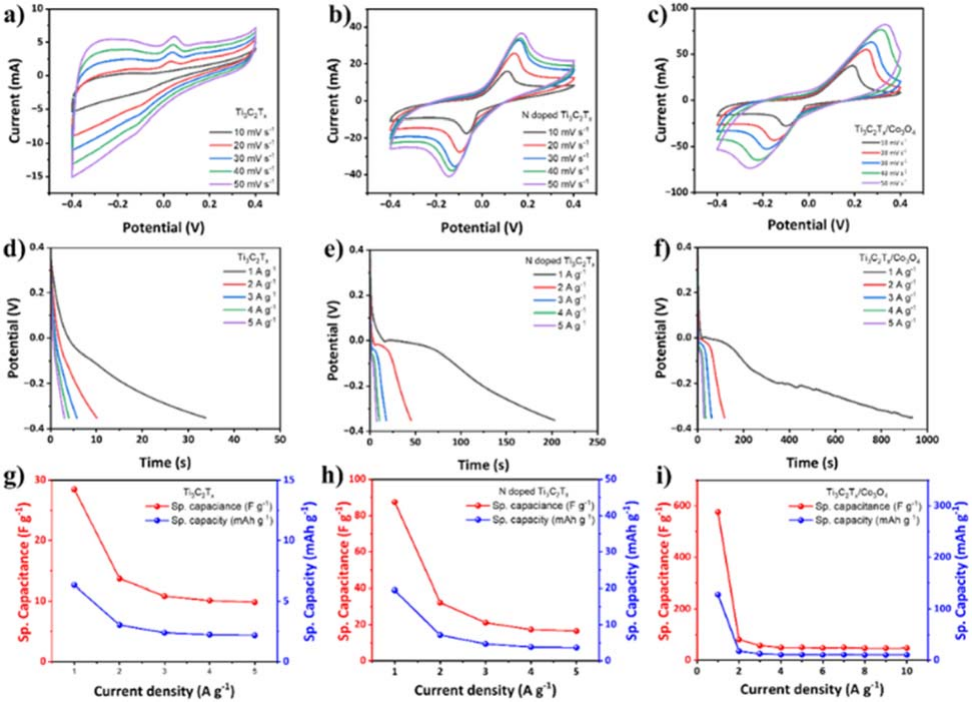
(a)–(c) CV curves of different electrodes at 10 mV s^−1^ scan rate, (d)–(f) discharge curves of different electrodes from GCD analysis at 1 A g^−1^ current density, and (g)–(i) specific capacity and specific capacitance in terms of area and mass.

At scan rates ranging from 10 to 50 mV s^−1^, the CV curves for Ti_3_C_2_T_*x*_, N-Ti_3_C_2_T_*x*_, and Ti_3_C_2_T_*x*_/Co_3_O_4_ electrodes have been obtained and are presented in Fig. 7(a)–(c). Based on the observations of the changes in redox peaks for the electrodes, the functional groups were more affected in a favorable manner by the Ti_3_C_2_T_*x*_/Co_3_O_4_ electrode deposited on CC compared to Ti_3_C_2_T_*x*_ and N-Ti_3_C_2_T_*x*_ electrodes. CV curves of Ti_3_C_2_T_*x*_/Co_3_O_4_ exhibit a handful of distinct redox peaks, which indicate the occurrence of pseudocapacitance. This pseudocapacitance was suitable with CV result when redox peak at −0.3 V stopping. In addition, the Ti_3_C_2_T_*x*_/Co_3_O_4_ electrode demonstrates a longer discharge time compared to other electrodes, suggesting a higher specific capacitance. The CV profile of the electrode in Fig. 7(c) maintains a consistent shape even at higher scan rates. This indicates that the electrode exhibits exceptional rate performance, which may be attributed to its high stability and wettability. All CV curves exhibit distinct redox peaks, which are predominantly linked to the faradaic redox processes. The trend indicating higher anodic and cathodic peak intensities at increased scan rates suggests efficient charge transfer and minimal resistance at the electrode–electrolyte interface. At lower scan rates, redox reactions that rely on proton (H^+^) insertion/desertion pathways from the electrolyte facilitate the diffusion of ions from the electrolyte into the accessible active sites in the electrode. Due to the interaction between hydronium ions (protons H^+^) and –O, bonding and debonding occur. The processes of bonding and debonding are reversible, thereby enabling the reversibility of the Ti valence state. Therefore, the electrolyte is expected to demonstrate a pseudocapacitive effect during cyclic voltammetry measurements. With an increase in the scan rate, the anodic peak moves to a more positive potential, while the cathodic peak moves to a more negative potential. This shift is attributed to the insufficient time available for ions to intercalate into the electrode.^63^ In comparison to bare CC and Ti_3_C_2_T_*x*_/CC, the peak current has increased as the oxidation current density improved. The discharge behavior of individual electrodes was measured at current densities ranging from 1 to 5 A g^−1^, as given in Fig. 7(d)–(f). The discharge time for the pristine Ti_3_C_2_T_*x*_ electrode was approximately 35 seconds, whereas the N-Ti_3_C_2_T_*x*_ electrode achieved a discharging time of up to 210 seconds. While for the hybrid composite electrode Ti_3_C_2_T_*x*_/Co_3_O_4_, the discharging time extended to nearly 950 seconds. Similarly, for comparison, N-Ti_3_C_2_T_*x*_/Co_3_O_4_ CV analysis has been carried out as well. Electrochemical analysis *i.e.* CV and GCD of the N-Ti_3_C_2_T_*x*_/Co_3_O_4_ electrode applied on carbon cloth is given in Fig. S11.† A detailed discussion is given in the ESI† for N-Ti_3_C_2_T_*x*_/Co_3_O_4_.

For the Ti_3_C_2_T_*x*_/Co_3_O_4_ hybrid composite, applied as an anode material on carbon cloth using a 1 M H_2_SO_4_ electrolyte, the working mechanisms at positive and negative potentials involve both electrochemical double-layer formation and faradaic reactions, attributable to the pseudocapacitive properties of the electrode materials.

Fig. 7(f) shows the galvanostatic discharge of the Ti_3_C_2_T_*x*_/Co_3_O_4_ hybrid composite sample at difference scan rates. To determine the specific capacitances and capacity of charge storage, the following equations were used:1
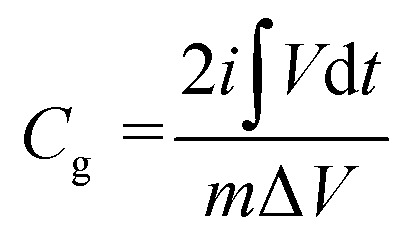
2
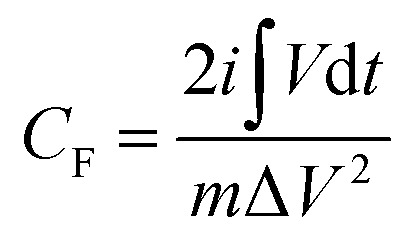
where *C*_g_ is the gravimetric capacity (A h g^−1^), *C*_F_ is the faradaic capacitance (F g^−1^), *i* is the current applied (A), 
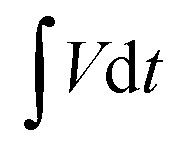
 is the integral area of the discharge curve (V s), *m* is the mass of the active material, and Δ*V* is the voltage difference in the discharge (V).

The specific capacitance values for each current density, obtained from the GCD curves, are shown in Fig. 7(g)–(i). At a current density of 1 A g^−1^, the Ti_3_C_2_T_*x*_/Co_3_O_4_ hybrid composite applied as an electrode has attained a maximum specific capacitance of 576.7 F g^−1^. While pristine Ti_3_C_2_T_*x*_ and N-Ti_3_C_2_T_*x*_ have attained the maximum specific capacitance of 28.5 F g^−1^ and 87.5 F g^−1^ at a current density of 1 A g^−1^, respectively.

## 7. Storage mechanism in the Ti_3_C_2_T_*x*_/Co_3_O_4_ hybrid composite

Regarding ion adsorption/desorption, the reaction mechanism is usually complex, owing to the different metal oxide redox species. Nonetheless, the most probable charge storage processes during anodic and cathodic sweeps have been summarized here. Ti_3_C_2_T_*x*_ being a 2D material with a layered structure, most of the charge storage is of electrochemical double-layer capacitance (EDLC) nature, due to its high surface area and enhanced conductivity. In the case of EDLC charge storage, the charges are stored electrostatically. Ti_3_C_2_T_*x*_ also exhibits pseudocapacitive behavior due to the presence of functional groups (*e.g.*, –OH, –O, and –F) on its surface. Cations (H^+^) from the H_2_SO_4_ electrolyte are attracted to the negatively charged Ti_3_C_2_T_*x*_ surface during anodic sweep. While during cathodic sweep, the H^+^ is de-intercalated or desorbed from the electrode material, and electrons are accepted from the external circuit. Likewise, oxidation and reduction of the surface attached functional groups occur during respective cycles, contributing to pseudocapacitance.^64,65^

In the case of the Ti_3_C_2_T_*x*_/Co_3_O_4_ hybrid composite, the high surface area and conductivity of Ti_3_C_2_T_*x*_ combine with the pseudocapacitive behavior of Co_3_O_4_. During anodic sweep, cations (H^+^) are attracted to the electrode surface and oxidation of Co^2+^ to Co^3+^ or Co^3+^ to Co^4+^ occurs, contributing to pseudocapacitance. Likewise, during cathodic sweep, cations (H^+^) are de-intercalated or desorbed from the electrode material. Reduction of Co^3+^ to Co^2+^ or Co^4+^ to Co^3+^ occurs, contributing to pseudocapacitance. For further exploring the effect of functional groups on the pseudocapacitance occurrence, FTIR analysis was carried out for pristine Ti_3_C_2_T_*x*_, Co_3_O_4_ and the hybrid composite Ti_3_C_2_T_*x*_/Co_3_O_4_. Detailed comparative explanation of the FTIR spectroscopy findings is given in Fig. S10.†

The Ti_3_C_2_T_*x*_ layered exfoliated structure facilitates efficient ion transport to the Co_3_O_4_ sites, which is crucial for electrochemical reactions, resulting in rapid charge and discharge cycles. While effective electron transport to Co_3_O_4_ nanoparticles is ensured by Ti_3_C_2_T_*x*_ superior electrical conductivity. This synergy of the hybrid composite enhances redox processes through reducing the internal resistance and improving the rate capability. In addition to this, the robust contact between Ti_3_C_2_T_*x*_ and Co_3_O_4_ nanoparticles maintains the structural integrity of the hybrid composite during the volumetric changes of charging and discharging.^66^

During charging, electrons are transferred from the hybrid composite Ti_3_C_2_T_*x*_/Co_3_O_4_ anode to the cathode. Concurrently, sulfate ions (SO_4_^2−^) from the H_2_SO_4_ electrolyte migrate towards the Ti_3_C_2_T_*x*_ layers, forming an electrical double layer at the electrode/electrolyte interface.^67^ The SO_4_^2−^ ions align near the positively charged surface of the Ti_3_C_2_T_*x*_/Co_3_O_4_ anode. Similarly, during charging at the negative electrode, hydrogen ions (H^+^) from the electrolyte are attracted towards its surface. An electric double layer forms at the electrode–electrolyte interface, with the H^+^ ions aligning near the negatively charged electrode surface. Additionally, pseudocapacitive behavior involves the following faradaic redox reactions:^68–71^

3 Co^2+^ ⇌ Co^3+^ + e^−^

4 Ti_3_C_2_O_*x*_(OH)_*y*_F_*z*_ + 1/2*x*e^−^ + 1/2*x*H^+^ ⇌ Ti_3_C_2_O_1/2*x*_(OH)_*y*+1/2*x*_F_*z*_

**Fig. 8:**
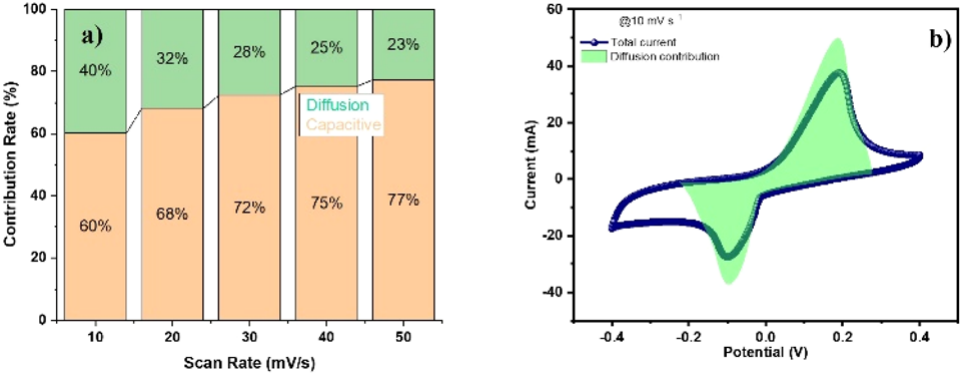
(a) Contribution ratios of capacitances from the pseudocapacitive controlled reaction and diffusion-controlled reaction at scan rates of 10 to 50 mV s^−1^ and (b) the kinetic analysis of the pseudocapacitive and diffusion parts at a scan rate of 10 mV s^−1^.

Additionally, in regard to the kinetic analysis aspects of charge storage mechanisms, it has involved the consideration of both the capacitive effects (*k*_1_*ν*) and the diffusion-controlled contributions (*k*_2_*ν*^0.5^) in relation to the voltage and response current.^72^ Respective percentile contributions from the Ti_3_C_2_T_*x*_/Co_3_O_4_ hybrid composite applied as the anode material was calculated using Dunn's method, at different potential scan rates *i.e.* 10–50 mV s^−1^ and plotted, as shown in Fig. 8(a). Respective contributions were determined by analyzing the CV curves presented in Fig. 8(a), according to Dunn's method:^73,74^

5 *i*(ν) = *k*_1_*ν* + *k*_2_*ν*^0.5^

or

6 *i*(ν)/*ν*^0.5^ = *k*_1_*ν*^0.5^ + *k*_2_

where *ν* is the scan rate and *i* is the current. The slope of the fitting line is represented by *k*_1_ and the intercept by *k*_2_, respectively, in the above equations. The quantitative study indicates that the CV curves at 10 mV s^−1^ have two sections capacity, as shown in Fig. 8(b). Fig. 8(b) shows a diffusion-controlled portion in the light green area and a capacitive-controlled region in the remaining area. As a result, at 10 mV s^−1^ and 50 mV s^−1^, respectively, the diffusion-controlled portion of the total charge stored was 40% and 23%. It is shown that when the sweep rate increased from low to high, the diffusion-controlled proportions steadily dropped.

## 8. Charge transfer mechanism investigation by EIS measurement

**Fig. 9:**
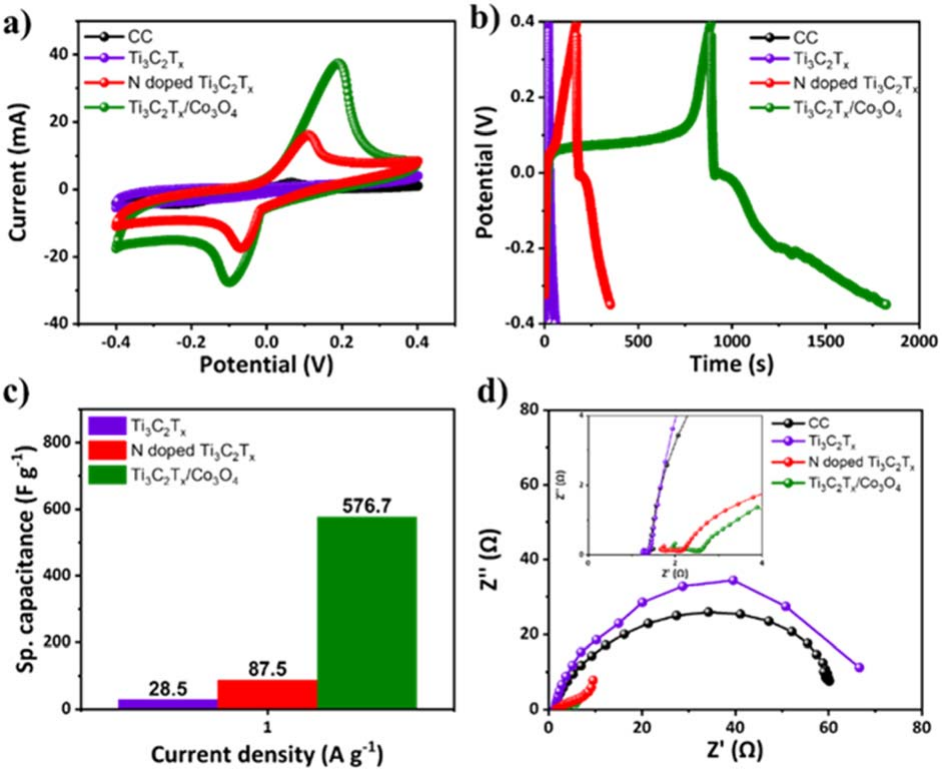
CC, Ti_3_C_2_T_*x*_, N-Ti_3_C_2_T_*x*_ and Ti_3_C_2_T_*x*_/Co_3_O_4_ electrodes: (a) CV curves at 10 mV s^−1^ scan rate, (b) discharge curves from GCD analysis at 1 A g^−1^ current density, (c) comparison of specific capacitance of Ti_3_C_2_T_*x*_, N-Ti_3_C_2_T_*x*_ and Ti_3_C_2_T_*x*_/Co_3_O_4_ electrodes, and (d) EIS spectrum analysis of CC, Ti_3_C_2_T_*x*_, N-Ti_3_C_2_T_*x*_ and Ti_3_C_2_T_*x*_/Co_3_O_4_.

For further elaboration and to make it easy for the researcher community to understand the charge transfer mechanism, CV and GCD curves at a current density of 1 A g^−1^ for CC and Ti_3_C_2_T_*x*_, N-Ti_3_C_2_T_*x*_ and the Ti_3_C_2_T_*x*_/Co_3_O_4_ hybrid composite were recorded, and the plots are shown in Fig. 9(a) and (b). Similarly, the obtained maximum specific capacitance values were plotted and compared, as shown in Fig. 9(c). The charge transfer mechanism of Ti_3_C_2_T_*x*_, N-Ti_3_C_2_T_*x*_ and Ti_3_C_2_T_*x*_/Co_3_O_4_ hybrid composite electrodes was investigated by conducting Electrochemical Impedance Spectroscopy (EIS) measurement. The obtained results for each electrode are shown in Fig. 9(d). EIS measurements provided further insights and understanding of the charge transfer mechanism. At higher frequencies, a smaller semicircle is observed for Ti_3_C_2_T_*x*_, indicating faster charge transfer and lesser resistance. While at lower frequencies, a vertical line is observed, which is an indication of optimal capacitive behavior and effective ion diffusion within Ti_3_C_2_T_*x*_. For N-Ti_3_C_2_T_*x*_, the EIS measurement has shown a bit flattened and broadened semicircle at higher frequencies compared to pristine Ti_3_C_2_T_*x*_, which is an indication that N-doping of Ti_3_C_2_T_*x*_ has slightly enhanced the charge transfer resistance in the electrolyte. This behavior shown in the EIS plot indicates the charge transfer limitations at the interface of N-Ti_3_C_2_T_*x*_/H_2_SO_4_ (electrode/electrolyte) for redox reactions. The flattened and broadened semicircle for N-Ti_3_C_2_T_*x*_, relative to pristine Ti_3_C_2_T_*x*_, indicates an increase in charge transfer resistance, potentially due to lattice distortions of Ti_3_C_2_T_*x*_ due to nitrogen incorporation. At intermediate and low frequencies, the vertical line obtained is more pronounced compared to the pristine Ti_3_C_2_T_*x*_. This behavior is an indication of enhanced capacitive behavior of N-Ti_3_C_2_T_*x*_ due to diffusion-controlled processes and pseudocapacitance. This has possibly occurred due to the increased number of electroactive sites and surface wettability.

The EIS spectra acquired for the Ti_3_C_2_T_*x*_/Co_3_O_4_ hybrid composite exhibit the complementary and synergistic effects of the two materials. A somewhat more flattened and broadened semicircle than pristine Ti_3_C_2_T_*x*_ and N-Ti_3_C_2_T_*x*_ has been observed at higher frequencies. It has been observed that due to incorporation of Co_3_O_4_ NPs pseudocapacitance through redox reactions manifested Warburg impedance in the mid-frequency range and furthermore showed a transitioning into a sloping line at lower frequencies. This characteristic indicates an enhancement in energy storage capacity due to combined double-layer capacitance and pseudocapacitance.

## 9. Conclusions

Successful synthesis of Ti_3_C_2_T_*x*_, N-doped Ti_3_C_2_T_*x*_, and Ti_3_C_2_T_*x*_/Co_3_O_4_ hybrid composite materials was achieved herein. Co_3_O_4_ nanoparticles were successfully synthesized using a novel device, microplasma discharge reactor, which is an eco-friendlier synthesis method. The synthesized materials were then employed as electrodes, using a slurry deposition technique involving the binder PVDF and Super P on carbon cloth. After careful analysis of Ti_3_C_2_T_*x*_, N-Ti_3_C_2_T_*x*_, and the Ti_3_C_2_T_*x*_/Co_3_O_4_ hybrid composite (4 : 1), it was noted that the Ti_3_C_2_T_*x*_/Co_3_O_4_ hybrid composite exhibited improved electrical conductivity. The incorporation of Co_3_O_4_ nanoparticles between the Ti_3_C_2_T_*x*_ nanosheets, which prevented restacking, is the reason for enhancement in performance. The Ti_3_C_2_T_*x*_/Co_3_O_4_ hybrid composite electrode showcased a remarkable specific capacitance of 576.7 F g^−1^ and a specific capacity of 128 A h g^−1^ when analyzed at a current density of 1 A g^−1^. The specific capacitance was compared to the pristine Ti_3_C_2_T_*x*_ electrode (28.5 F g^−1^), and it showed a significant increase of 95.06% (20.23 times). The Ti_3_C_2_T_*x*_/Co_3_O_4_ hybrid composite showed outstanding electrochemical performance, demonstrating its extensive potential for use in cutting-edge energy storage systems. To improve the electrochemical performance of Ti_3_C_2_T_*x*_ and provide new perspectives for diverse applications, it is essential to achieve an ideal balance of Co_3_O_4_ nanoparticles.

## Author contributions

Lan Nguyen: synthesis of materials, electrochemical analysis, data curation, manuscript writing. Adnan Ali: conception, design, analysis, data curation, writing – original draft. Sosiawati Teke: synthesis of Co_3_O_4_ NPs *via* MPR, characterization. Roshan Mangal Bhattarai: assisted in electrochemical analysis, data curation. Avik Denra and Oai Vu Quoc: review and editing of the manuscript. Young Sun Mok: supervision, resources, funding acquisition, validation.

## Conflicts of interest

The authors declare that they have no known competing financial interests or personal relationships that could have appeared to influence the work reported in this paper.

## Supplementary Material

NA-007-D4NA01024H-s001

## Data Availability

The data supporting this article have been included as part of the ESI.†
