# Supplementary material for: Electrochemical investigation of Ti3C2Tx (MXene), N-Ti3C2Tx, and the Ti3C2Tx/Co3O4 hybrid composite deposited on carbon cloth for use as anode materials in flexible supercapacitors
Source: Nanoscale Adv. 2025 May 27;7(17):5313–22. doi: 10.1039/d4na01024h (PMC12282667; doi:10.1039/d4na01024h)
Supplement: NA-007-D4NA01024H-s001 [file NA-007-D4NA01024H-s001.pdf]

Supplementary Information (SI) for Nanoscale Advances.
This journal is © The Royal Society of Chemistry 2025

Supplementary Data

Electrochemical Investigation of $Ti_3C_2T_x$ (MXene), N-$Ti_3C_2T_x$, and $Ti_3C_2T_x/Co_3O_4$ Hybrid Composite Deposited on Carbon Cloth for use as Anode Materials in Flexible Supercapacitors

Lan Nguyen,[a] Adnan Ali,[a*] Sosiawati Teke,[a,b] Roshan Mangal Bhattarai,[a] Avik Denra,[a] Oai Vu Quoc,[a] and Young Sun Mok[a*]

[a] Department of Chemical Engineering, Jeju National University, Jeju 63243, Republic of Korea

[b] Department of Physics, Halu Oleo University, Kendari-93132, Republic of Indonesia.

E-mail: adali@jejunu.ac.kr; smokie@jejunu.ac.kr; Fax: +82-64-755-3670; Tel: +82-64-754-3682

Microplasma Discharge Reactor Mechanism:

One way is to synthesize nanoparticles by plasma contact with a liquid interface. Plasma is an ionized gas that contains photons, energetic electrons, ions, radicals, and neutral gas molecules in various excited states. When plasma interacts with a liquid surface (plasma-liquid interface), reactive species like O^-, OH^-, H_2O_2, and UV light are produced and act as reducing agents. Using a plasma-liquid interface can minimize the use of potentially hazardous chemicals in the nanoparticle synthesis process, which makes it more environmentally friendly compared to conventional techniques.[1-3] Microplasma discharge reactors, a type of plasma with microscale geometry, is still being researched for potential use in nanomaterial synthesis. Microplasma delivers products with high homogeneity.

The mechanism of nanomaterial synthesis in a microplasma discharge reactor involves several key steps such as nucleation, growth, annealing & characterization, termination, and collection, presented in Figure S1 (a).. The process begins with the generation of a microplasma between electrodes in a reactor filled with a precursor gas or a mixture of gases. The high-energy environment within the microplasma leads to the dissociation of gas molecules and the generation of reactive species (e.g., atoms, ions, radicals). These reactive species undergo supersaturation in the localized region of the microplasma. When the concentration of these species exceeds the saturation limit, nucleation occurs, leading to the formation of tiny clusters. These clusters are the precursors to nanomaterials. The high-energy electrons and ions in the plasma transfer energy to the growing nanomaterials, leading to annealing processes that improve the crystallinity and modify the phase composition of the nanomaterials. After this, The nanomaterials can be rapidly quenched or cooled by removing them from the plasma region, which helps preserve their size, shape, and phase composition, histogram of the controlled size Co_3O_4 nanoparticles has been given in Figure S1(b). Finally, the synthesized nanomaterials are collected.

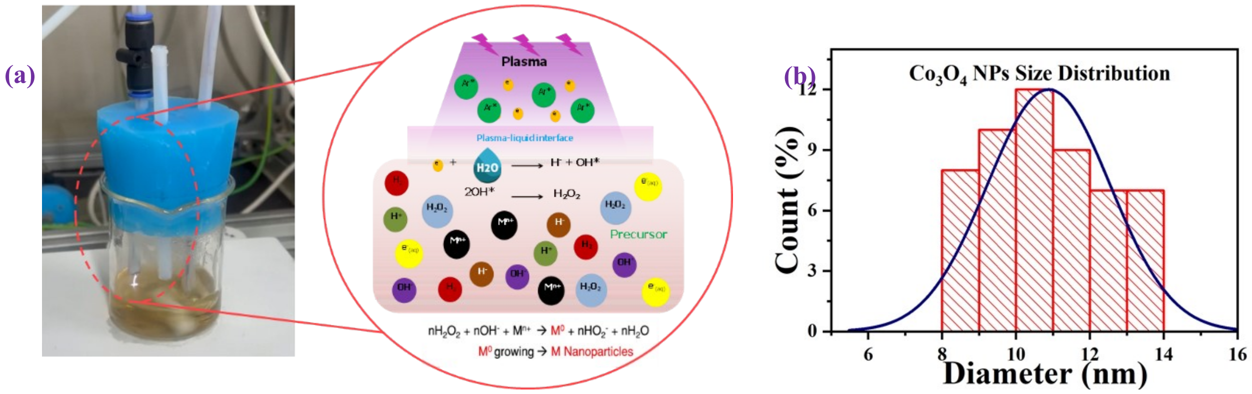

Figure S1. (a) Microplasma Discharge Reactor and Mechanism, (b) Histogram of Co_3O_4 NPs size distribution

Per Gram of Co_3O_4 Nanoparticles synthesis cost via MPR Technique:

As MPR technique is cost-effective and the estimated cost for synthesizing 1 gram of Co_3O_4 nanoparticles:

Chemical/Gas	Price	Per synthesis	Cost per synthesis
$Co(NO_3)_2.6H_2O$	\$108.35 (100 g)	0.073 g	\$0.079
Argon gas (99.996%) (1.5 L/min)	\$47 (6 m^3)	0.0375 m^3	\$0.25
**Total**			**\$0.329**

The power output of the microplasma reactor is calculated using this equation:

$$P = f \times \int P(t)dt = f \times \int V(t) \times I(t)dt$$

From this, the actual power was found to be 6.03 W.

For a synthesis duration of 25 min (or 0.4167 hours), the energy consumption is:

Energy = Power x Time = 6.03 x 0.4167 = 0.0025 kWh

Assuming an electricity rate of \$0.15 per kWh, the electricity cost per synthesis is:

Electricity cost = 0.15 x 0.0025 = \$0.000375

After the calcination process, each synthesis yields approximately 0.047 g of Co_3O_4 nanoparticles.

Total cost per synthesis:

Total cost = 0.079 + 0.25 + 0.000375 = \$0.329

Hence, to produce 1 gram of Co_3O_4 nanoparticles, cost per gram is:

Cost per gram = 0.329/0.047 = \$7

Morphological Characterization of Ti_3AlC_2 (MAX Phase):

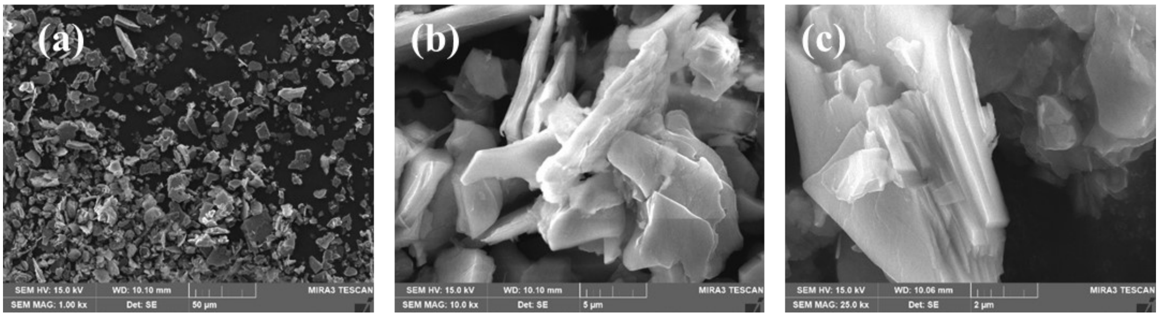

Figure S2. (a-c) FESEM Analysis of Ti_3AlC_2

Morphological Characterization of $Ti_3C_2T_x$ (MXene):

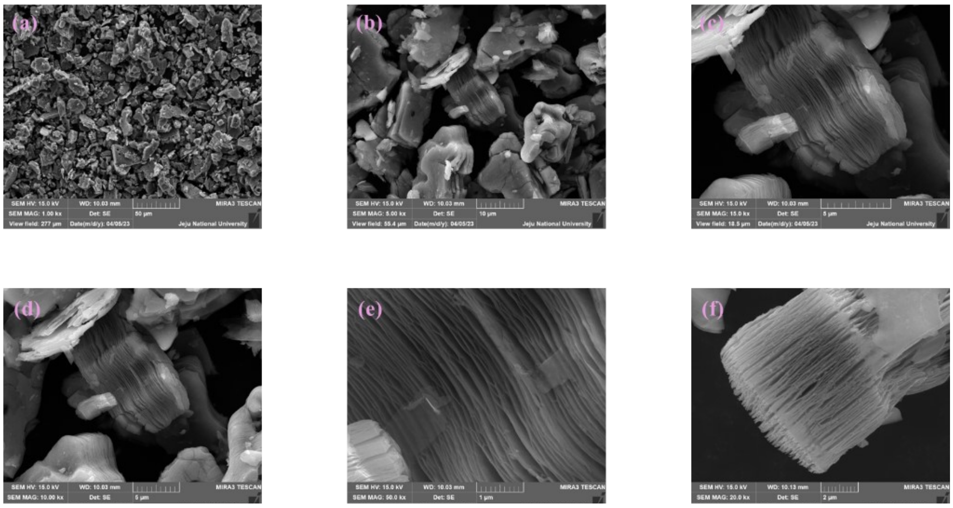

Figure S3. (a-f) FESEM images of $Ti_3C_2T_x$ at different resolutions after etching and exfoliation

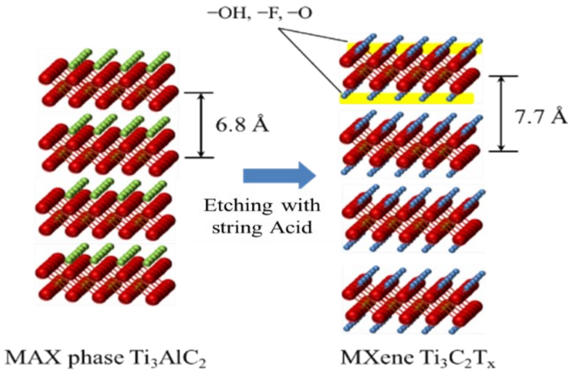

Figure S4. Schematic representation of MAX phase before and after HF etching.

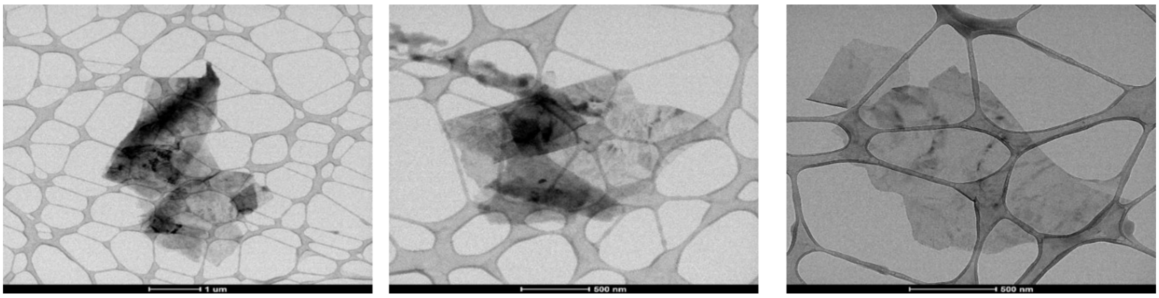

Figure S5. HRTEM images of $Ti_3C_2T_x$

Energy Dispersive Spectroscopy Analysis of $Ti_3C_2T_x$:

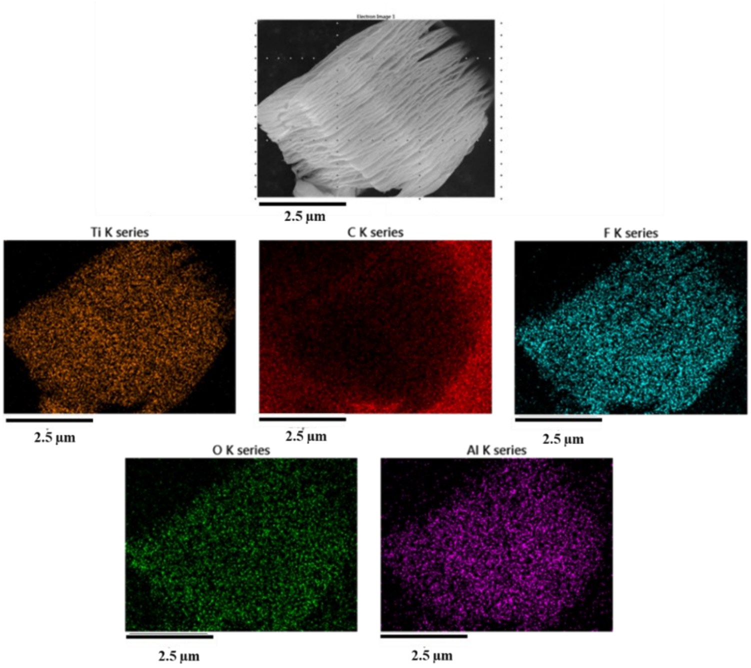

Figure S6. EDS mapping of Ti_3C_2T for elemental analysis

Energy Dispersive Spectroscopy Analysis of $Ti_3C_2T_x/Co_3O_4$ nanocomposite:

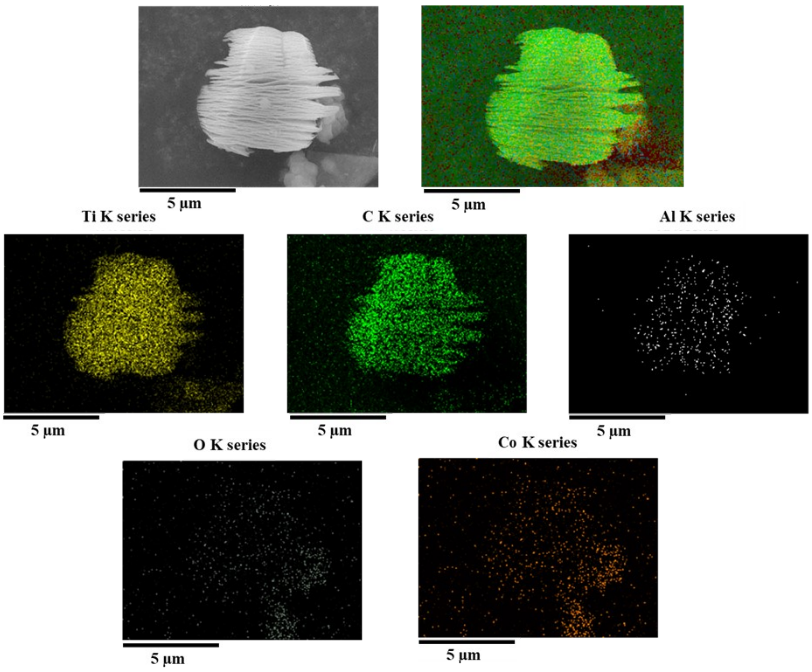

Figure S7. EDS mapping and elemental analysis of of $Ti_3C_2T_x/Co_3O_4$ hybrid nanocomposite

X-Ray Diffraction and XPS Analysis of Nitrogen Doped $Ti_3C_2T_x$:

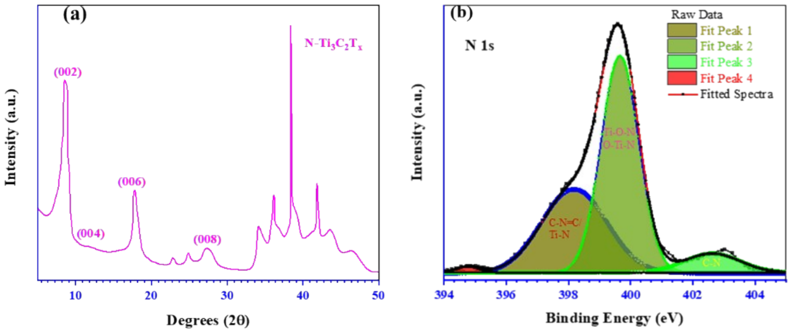

Figure S8. (a) XRD Pattern and (b) High Resolution deconvulated XPS Spectrum of N-$Ti_3C_2T_x$

Raman Spectroscopy Analysis:

Raman spectroscopy has carried out for the $Ti_3C_2T_x$, Co_3O_4 and $Ti_3C_2T_x/Co_3O_4$ nanocomposite, presented in Figure S9, to elucidate the vibrational modes and structural dynamics of these. In the case of $Ti_3C_2T_x$, a layered two-dimensional material, the identified peaks at 202 cm^{-1}, 286 cm^{-1}, 395 cm^{-1}, 567 cm^{-1}, 628 cm^{-1}, and 736 cm^{-1} are indicative of specific vibrational modes that are modulated by its surface functional groups, including -OH, -O, and -F. The modes encompass E_g vibrations, indicative of in-plane movements of Ti and C atoms, and F_{2g} vibrations, which pertain to out-of-plane interactions among the layers and the functional groups.[4, 5] In case of Co_3O_4 synthesized nanoparticles, Raman spectroscopy analysis displayed distinct peaks at 485 cm^{-1}, 527 cm^{-1}, 633 cm^{-1}, and 704 cm^{-1}, which are indicative of its spinel structure. The vibrational modes encompass F_{2g}, which are associated to the symmetric stretching of Co-O bonds located in tetrahedral sites, and E_g, which is associated with the bending vibrations of Co-O bonds situated in octahedral sites.[6] These features are essential for comprehending the structural properties that influence the catalytic and electrochemical behavior of Co_3O_4.

Within the $Ti_3C_2T_x/Co_3O_4$ nanocomposite, the Raman spectrum has revealed an additional peak at 179 cm^{-1}, in conjunction with signals from both $Ti_3C_2T_x$ and Co_3O_4. The distinctive peak indicates robust structural interactions between the two components, resulting in a composite material characterized by superior physical and chemical properties. The vibrational modes encompass E_{1g}, which signifies an increased interaction between $Ti_3C_2T_x$ and Co_3O_4, alongside a synthesis of E_g and F_{2g} modes derived from the constituent materials. The interactions yielded a synergistic effect, which has enhanced conductivity, catalytic activity, and various functional attributes of the $Ti_3C_2T_x/Co_3O_4$ nanocomposite.[7, 8]

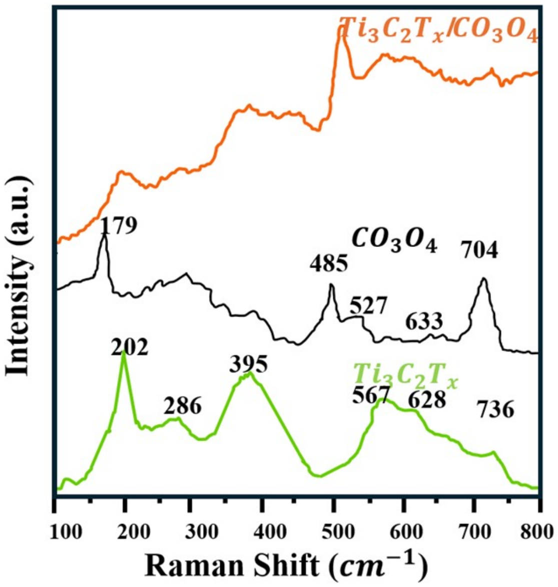

Figure S9. Raman Spectroscopy of the $Ti_3C_2T_x$, Co_3O_4 and $Ti_3C_2T_x/Co_3O_4$

FTIR Analysis for Functional Groups:

The FTIR spectrum of $Ti_3C_2T_x$, shown in Figure S10 has revealed distinct peaks that correspond to various functional groups, including Ti-C, Ti-O, Ti-O-Ti, C-F, C=O, and O-H. The presence of these functional groups facilitates rapid and reversible faradaic reactions at the electrode interface, which is a fundamental mechanism underlying pseudocapacitance.[9] The hydroxyl (O-H) and carbonyl (C=O) functional groups play a crucial role in facilitating redox reactions, thereby significantly improving the charge storage capacity of the material.[9] Nonetheless, $Ti_3C_2T_x$ exhibits a tendency to restack as a consequence of its layered structure, which constrains ion accessibility by diminishing the interlayer spacing available.

Similarly, the FTIR spectrum of Co_3O_4 was obtained, presented in Figure S10 and it has distinctly indicated the presence of Co-O bonds, which are typical of its spinel structural configuration. These bonds enable redox reactions that involve Co ions, which play a significant role in pseudocapacitance. Furthermore, the C=O bonds identified in the spectrum serve as active sites for ion adsorption and faradaic processes, thereby enhancing its charge storage capacity.[10] The distinctive architecture of Co_3O_4 nanoparticles enhances surface area and facilitates elevated electrochemical activity.

The $Ti_3C_2T_x/Co_3O_4$ nanocomposite has integrated the advantageous properties of both the materials, as demonstrated by its FTIR spectrum (presented in Figure S10) which reveals distinct peaks corresponding to Ti-C, Ti-O, Co-O, C=O, and O-H functional groups.[11, 12] As in this composite, Co_3O_4 nanoparticles are intercalated within the layers of $Ti_3C_2T_x$. This strategic configuration inhibits the restacking of the $Ti_3C_2T_x$ layers, thus preserving interlayer spacing and markedly enhancing the accessible surface area. This configuration not only facilitates enhanced ion transport pathways but also generates an increased number of active sites for faradaic reactions. The nanocomposite demonstrates superior pseudocapacitive behavior, increased conductivity, and an augmented charge storage capacity when compared to its constituent elements.

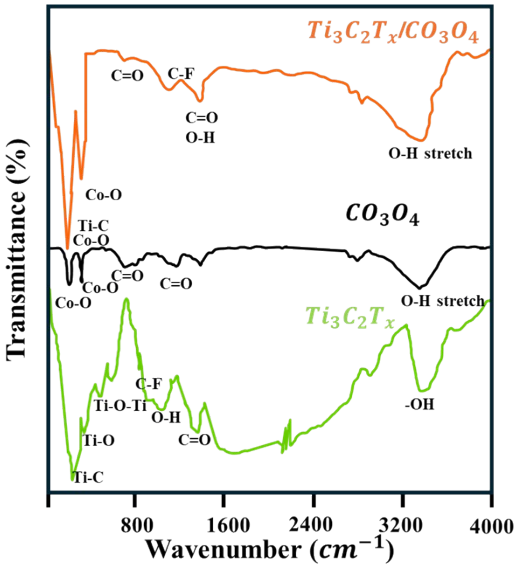

Figure S10. FTIR Analysis of the $Ti_3C_2T_x$, Co_3O_4 and $Ti_3C_2T_x/Co_3O_4$

Carbon cloth and N-$Ti_3C_2T_x/Co_3O_4$ Electrochemical Analysis:

The CV curves for carbon cloth and N-$Ti_3C_2T_x/Co_3O_4$ electrode has been obtained at different scan rates ranging presented in Figure S11 (a, b). Based on the observations of the changes in redox peaks for the electrode, it has been understood that due to N doping the interlayer spacing of the $Ti_3C_2T_x$ layers might have increased more. Due

to this, more of the Co_3O_4 nanoparticles entered between the $Ti_3C_2T_x$ layers and settled there. This has affected the overall performance of the applied electrode, because the surface area and active sites dropped enormously. This has been confirmed by the CV and GCD of the N-$Ti_3C_2T_x$/Co_3O_4 electrode deposited on CC, as shown in Figure S11 (c). It has observed that GCD of the N-$Ti_3C_2T_x$/Co_3O_4 is better than $Ti_3C_2T_x$ and N-$Ti_3C_2T_x$ but much lesser compared to $Ti_3C_2T_x$/Co_3O_4 electrodes.

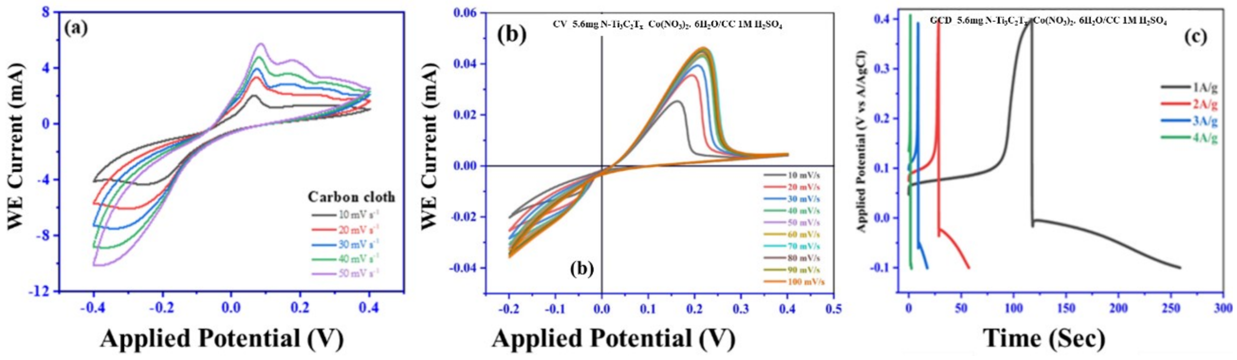

Figure S11. (a-b) Cyclic Voltammetry measurement of Carbon Cloth and N-$Ti_3C_2T_x$/Co_3O_4 at different voltage scan rates, (c) GCD of N-$Ti_3C_2T_x$/Co_3O_4.

Reference:

1. P. J. Bruggeman, M. J. Kushner, B. R. Locke, J. G. E. Gardeniers, W. G. Graham, D. B. Graves, R. C. H. M. Hofman-Caris, D. Maric, J. P. Reid, E. Ceriani, D. Fernandez Rivas, J. E. Foster, S. C. Garrick, Y. Gorbanev, S. Hamaguchi, F. Iza, H. Jablonowski, E. Klimova, J. Kolb, F. Krcma, P. Lukes, Z. Machala, I. Marinov, D. Mariotti, S. Mededovic Thagard, D. Minakata, E. C. Neyts, J. Pawlat, Z. L. Petrovic, R. Pflieger, S. Reuter, D. C. Schram, S. Schröter, M. Shiraiwa, B. Tarabová, P. A. Tsai, J. R. R. Verlet, T. von Woedtke, K. R. Wilson, K. Yasui and G. Zvereva, *Plasma Sources Science and Technology*, 2016, **25**, 053002.
2. R. Grinham and A. Chew, *Applied Science and Convergence Technology*, 2017, **26**, 95-109.
3. Q. Chen, J. Li and Y. Li, *Journal of Physics D: Applied Physics*, 2015, **48**, 424005.
4. S. Adomaviciute-Grabusove, A. Popov, S. Ramanavicius, V. Sablinskas, K. Shevchuk, O. Gogotsi, I. Baginskiy, Y. Gogotsi and A. Ramanavicius, *ACS Nano*, 2024, **18**, 13184-13195.
5. A. Sarycheva and Y. Gogotsi, *Chemistry of Materials*, 2020, **32**, 3480-3488.
6. B. Ren, G.-K. Liu, X.-B. Lian, Z.-L. Yang and Z.-Q. Tian, *Analytical and Bioanalytical Chemistry*, 2007, **388**, 29-45.
7. Y. Wang, X. Wei, X. Hu, W. Zhou and Y. Zhao, *Catalysis Letters*, 2019, **149**.
8. M. Benchakar, T. Bilyk, C. Garnero, L. Loupias, C. Morais, J. Pacaud, C. Canaff, P. Chartier, S. Morisset, N. Guignard, V. Mauchamp, S. Celerier and A. Habrioux, *Advanced Materials Interfaces*, 2019, DOI: 10.1002/admi.201901328.
9. Z. Liu, H. Lv, Y. Xie, J. Wang, J. Fan, B. Sun, L. Jiang, Y. Zhang, R. Wang and K. Shi, *Journal of Materials Chemistry A*, 2022, **10**.
10. A. L. Bhatti, A. Tahira, I. A. Halepoto, S. Kumar, Z. A. Ujjan, A. Nafady, N. A. Shama, E. Dawi, A. Ashames, L. Saleem, M. Tonezzer, A. Infantes-Molin and Z. H. Ibupoto, *Journal of Electronic Materials*, 2024, **53**, 1012-1025.
11. N. Liu, N. Lu, H. Yu, S. Chen and X. Quan, *Chemosphere*, 2020, **246**, 125760.
12. x. Hongyan, Z. Hai, J. Diwu, Q. Zhang, L. Gao, D. Cui, J. Zang, J. Liu and C. Xue, *Journal of Nanomaterials*, 2015, **2015**, 1-8.
